# A kinetic model of multiple phenotypic states for breast cancer cells

**DOI:** 10.1038/s41598-017-10321-1

**Published:** 2017-08-29

**Authors:** Kang Qiu, Kai-fu Gao, Li-jian Yang, Zhao-kang Zhang, Ran Wang, Hui-shu Ma, Ya Jia

**Affiliations:** 1 Institute of Biophysics and Department of Physics, Central China Normal University, Wuhan, 430079 China; 2 Department of Mathematics and Physics, Xuzhou Medical University, Xuzhou, 221004 China

## Abstract

Quantitative modeling of microscopic genes regulatory mechanisms in an individual cell is a crucial step towards understanding various macroscopic physiological phenomena of cell populations. Based on the regulatory mechanisms of genes *zeb1* and *cdh1* in the growth and development of breast cancer cells, we propose a kinetic model at the level of single cell. By constructing the effective landscape of underlying stationary probability for the genes expressions, it is found that (i) each breast cancer cell has three phenotypic states (i.e., the stem-like, basal, and luminal states) which correspond to three attractions of the probability landscape. (ii) The interconversions between phenotypic states can be induced by the noise intensity and the property of phenotypic switching is quantified by the mean first-passage time. (iii) Under certain conditions, the probabilities of each cancer cell appearing in the three states are consistent with the macroscopic phenotypic equilibrium proportions in the breast cancer SUM159 cell line. (iv) Our kinetic model involving the TGF-β signal can also qualitatively explain several macroscopic physiological phenomena of breast cancer cells, such as the “TGF-β paradox” in tumor therapy, the five clinical subtypes of breast cancer cells, and the effects of transient TGF-β on breast cancer metastasis.

## Introduction

The regulation of cell phenotype decisions is critical for the survival of living cells. The clonal or stem cell was found with multiple phenotypic states, for example, the multiple states can arise in a cell with different gene expression states in *E. coli* ^1^. The cell state changes occur in response to microenvironmental signals and fluctuations^2–17^. The multiple phenotypic states also exist in a variety of cancer cells^18–24^, such as breast, colorectal cancers, etc.

Recent experimental observations^25^ demonstrated there are three mammary epithelial cell phenotypic states (i.e., the stem-like, basal, and luminal states) in human breast cancer cell lines (the primary tumors SUM159 and SUM149), and the subpopulations of cancer cells purified for a given phenotypic state return towards equilibrium proportions of three phenotypes over time. It was found that the phenomenon of phenotypic proportions in human breast cancer cell lines is not due to differential growth rates of cells in the basal, stem-like, or luminal state but rather to interconversion between the three states, and a Markov model in which breast cancer cells transit stochastically between states was proposed to explain those experimental observations.

The observed breast cancer populations^25^ are composed of a large number of cancer cells, although the cancer cells transition stochastically between three states, it is assumed that each cancer cell has the same gene regulatory pathways or kinetics of genes regulatory mechanisms *in vivo*. Understanding how the macroscopic phenotypic equilibrium proportions arise in each cancer cell and how the multiple states coexistence of an individual cell is mapped onto various macroscopic phenomena at the level of the whole cancer populations, implies that we ought to structure useful kinetic models of microscopic regulatory mechanisms at the single cell level. Therefore, the question of how the cell-state decisions of each cancer cell are made by genes regulatory mechanisms is critical outstanding. To our knowledge, however, the kinetic model of microscopic regulatory mechanisms for the multiple phenotypic states of an individual cancer cell is still unknown so far.

Although there are a large number of genes involved in the multiple phenotypic states of an individual cancer cell, a few key genes regulations might determine the cancer cell’s phenotype or invasion and metastasis, and the cancer cell’s response to microenvironmental signals (such as oestrogen, TGF-β, survival factors, cytokines and extracellular matrix)^26–28^. For example, genes *zeb1* (the transcription factor ZEB1) and *cdh1* (encoding the protein E-Cadherin) play a vital role in cancer cells developmental processes^29, 30^, especially in the epithelial-mesenchymal transition (EMT) process, which is a key developmental program that is often activated during cancer invasion and metastasis. Thus, interesting questions now arise: Can the macroscopic phenotypic equilibrium phenomena at the level of the whole breast cancer populations be understood by the multiple states coexistence of each cancer cell at the level of single cell? What is the kinetic model of the key genes regulatory mechanism in a cancer cell?

In this paper, based on the transcriptional regulatory mechanisms between two key genes (*zeb1* and *cdh1*) in the developmental process of breast cancer cells, we proposed a general kinetic model of the genes regulation mechanisms for each cancer cell, our results showed that each cancer cell also exists three phenotypic states (i.e., the stem-like, basal, and luminal states), and there are interconversions between the three phenotypic states. In order to quantify the properties of phenotypic transition (or switching) between states, a theoretical formula of mean first-passage time is derived. Most interestingly, our general kinetic model of genes regulation mechanisms at the single cell level could help one to understand some macroscopic physiological phenomena at the level of whole breast cancer cell population, such as the phenotypic equilibrium in subpopulations of breast cancer lines^25^, the “TGF-β paradox” in tumor therapy^31–41^, the five clinical subtypes of breast cancer cells^42, 43^, and the effects of transient TGF-β on cancer metastasis^44^.

The paper is arranged as follows. Firstly, a general kinetic model for the multiple phenotypic states of each breast cancer cell is proposed at the level of single cancer cell, and a theoretical formula of mean first-passage time for the phenotypic switching between states is derived by using an approximate Fokker-Planck equation. Secondly, we study the multiple phenotypic states coexistence and the phenotypic switching of an individual cancer cell. Then, the expression levels of genes *zeb1* and *cdh1* and the probabilities of an individual cancer cell appearing in three phenotypic states are compared with those of the human breast SUM159 line. Most interestingly, several clinical and therapeutic phenomena of breast tumors are qualitatively discussed by virtue of the general kinetic model. We end with the conclusions and discussions.

## General kinetic model of key genes regulations

### The stochastic kinetic model

**Figure 1:**
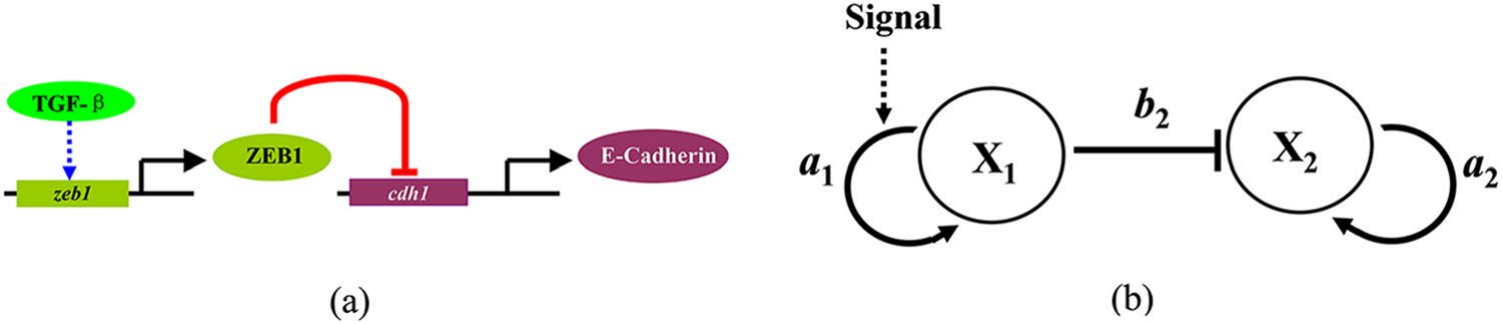
A schematic diagram of key genes regulations. (**a**) The microscopic regulatory mechanisms between genes *zeb1* and *cdh1* in breast cancer cells^24, 29, 30, 45^, where the expression of EMT-associated transcription factor ZEB1 can be induced by TGF-β signaling^51^. (**b**) A general kinetic model of the genes regulatory mechanisms, where *a*_1_ and *a*_2_ are the self-activation rates of genes *X*_1_ and *X*_2_, *b*_2_ is the strength of inhibition by the transcriptional factor of *X*_1_.

In the developmental process of breast cancer cells, it was found that the transcription factor ZEB1 can promote EMT through inhibiting the expression of gene *cdh1* (which encodes the adhesion protein E-Cadherin) as shown in Fig. 1(a) ^24, 29, 30, 45^. The E-Cadherin is a kind of transmembrane protein and essential for the stable cell-cell adhesion, and plays an important role in cellular development and cancer metastasis through modulating the EMT and the mesenchymal-epithelial transition (MET)^29, 30, 45^. The low expression of E-Cadherin (through allelic loss and methylation/hyper-methylation of 5’CpG sites of *cdh1*) can promote tumor metastasis and malignancy in the early stage of a tumor, the high expression of E-Cadherin can induce new tumors forming at distant organs in the late stage of the tumor^46–48^. The activation of *zeb1* induces the stem-like cells by inhibiting the expression of mir-200 family members which repress the stemness-associated factors such as SOX2 and KLF4^28, 49, 50^. The expression of EMT-associated transcription factors (such as SNAIL1, SNAIL2, ZEB1, ZEB2, and LEF1) can be induced by TGF-β signal^51^.

Above microscopic regulatory mechanisms of the key genes *zeb1* and *cdh1* in the developmental process of breast cancer cells can be described by a general regulatory model as shown in Fig. 1(b), where *X*_1_ represents the gene *zeb1*, and *X*_2_ represents the gene *cdh1*. In the deterministic description, the kinetic model of the genes regulatory mechanisms with a transcriptional negative regulation can be written as following ordinary differential equations in the dimensionless:

1 $$\frac{d{x}_{1}}{dt}={a}_{1}\frac{{x}_{1}^{n}}{{\theta }^{n}+{x}_{1}^{n}}+{b}_{1}-{k}_{1}{x}_{1}={F}_{1}({x}_{1})$$

2 $$\frac{d{x}_{2}}{dt}={a}_{2}\frac{{x}_{2}^{n}}{{\theta }^{n}+{x}_{2}^{n}}+{b}_{2}\frac{{\theta }^{n}}{{\theta }^{n}+{x}_{1}^{n}}-{k}_{2}{x}_{2}={F}_{2}({x}_{1},{x}_{2})$$

where *x*_1_ and *x*_2_ represent the expression level of genes *X*_1_ and *X*_2_, respectively.*a*_1_ and *a*_2_ are the self-activation rates of genes, *b*_1_ is the basal expression rate of *X*_1_, and *b*_2_ is the strength of inhibition by the transcriptional factor of *X*_1_. *k*_1_ and *k*_2_ are the self-degradation rates.*θ* represents the threshold which is the critical value needed for appreciable changes, and *n* is the Hill coefficient which controls the steepness of the sigmoidal function. The parameter values are *k*_1_ = *k*_2_ = 1.0, *θ* = 0.5, *n* = 4, *b*_1_ = 0.2, and *b*_2_ = 1.0 for simplicity. It is hypothesized that the transcriptional negative regulation on *X*_2_ by *X*_1_ and the self-activation of *X*_2_ do not simultaneously occur and the regulations in equation (2) follow an “or” rather than “and” logic^13^.

The regulations and expressions of genes are the intracellular random biochemical events^4–8, 10^, and the stochasticity plays an important role in regulating cell-state equilibria in subpopulations of cells^25^. In the stochastic description, the equations (1) and (2) are described by following stochastic differential equations:

3 $$\frac{d{x}_{1}}{dt}={F}_{1}({x}_{1})+{\xi }_{1}(t)$$

4 $$\frac{d{x}_{2}}{dt}={F}_{2}({x}_{1},{x}_{2})+{\xi }_{2}(t)$$

where *ξ*_1_(*t*) and *ξ*_2_(*t*) are Gaussion white noises with zero means and 〈*ξ*_1_(*t*)*ξ*_1_(*s*)〉 = 2*D*_1_ *δ*(*t* − *s*), 〈*ξ*_2_(*t*)*ξ*_2_(*s*)〉 = 2*D*_2_ *δ*(*t* − *s*). Here we consider a homogeneous and non-correlation situation *D*_1_ = *D*_2_ ≡ *D* which represents the total effect of intrinsic and extrinsic noises. Hence, the probability distribution *P*(*x*_1_, *x*_2_, *t*) of equations (3) and (4) obeys the Fokker-Planck equation^52–54^:

5 $$\frac{\partial P({x}_{1},{x}_{2},t)}{\partial t}=-\frac{\partial }{\partial {x}_{1}}[{F}_{1}({x}_{1})P({x}_{1},{x}_{2},t)]-\frac{\partial }{\partial {x}_{2}}[{F}_{2}({x}_{1},{x}_{2})P({x}_{1},{x}_{2},t)]+D(\frac{{\partial }^{2}}{\partial {x}_{1}^{2}}+\frac{{\partial }^{2}}{\partial {x}_{2}^{2}})P({x}_{1},{x}_{2},t)$$

In the equilibrium case, the stationary probability *P*_*st*_(*x*_1_, *x*_2_) of equation (5) represents the states distribution of cancer cells. An effective potential function *U*_*st*_(*x*_1_, *x*_2_) for nonequilibrium system is defined by the stationary probability:

6 $${U}_{st}({x}_{1},{x}_{2})=-\mathrm{ln}\,[{P}_{st}({x}_{{\rm{1}}},{x}_{2})]$$

Each minimum of the potential function *U*_*st*_(*x*_1_, *x*_2_) corresponds to one state (or phenotype) of a cancer cell. The phenotypic switching of a cancer cell means that the state of the cancer cell moves from one minimum of potential landscape to another.

### A formula of phenotypic switching

To quantify the properties of phenotypic switching between states in the case of multiple phenotypic states coexistence, one can calculate the escape time from one steady state of *U*_*st*_(*x*_1_, *x*_2_) to another. A rigorous definition of escape time out of *y*_1_ is provided by the mean first-passage time (MFPT) *τ* of the stochastic process *y*(*t*) to reach the point *y*_2_ with initial condition *y*(*t* = 0) = *y*_1_. This is given by^55, 56^

7 $$\tau ={\int }_{{y}_{1}}^{{y}_{2}}\frac{dy}{D(y){P}_{st}(y)}{\int }_{-\infty }^{y}{P}_{st}(k)dk.$$

If the noise *ξ*_1_(*t*) in equations (3) is neglected, the stationary expression level ${x}_{1}^{st}$ of gene *X*_1_ can be obtained by setting *dx*_1_/*dt* = 0, then one can substitute ${x}_{1}={x}_{1}^{st}\equiv {x}_{0}$ into equation (4), and

8 $${F}_{2}({x}_{0},{x}_{2})={a}_{2}\frac{{{x}_{2}}^{4}}{{\theta }^{4}+{{x}_{2}}^{4}}+{b}_{2}\frac{{\theta }^{4}}{{\theta }^{4}+{{x}_{0}}^{4}}-{k}_{2}{x}_{2}$$

which corresponds to the potential function:

9 $$\begin{array}{rcl}U({x}_{0},{x}_{2}) & = & \frac{{a}_{2}\theta }{2\sqrt{2}}[{\rm{arctg}}(\frac{{{x}_{2}}^{2}-{\theta }^{2}}{\sqrt{2}\theta {x}_{2}})-\frac{1}{2}\,\mathrm{ln}(\frac{{{x}_{2}}^{2}-\sqrt{2}\theta {x}_{2}+{\theta }^{2}}{{{x}_{2}}^{2}+\sqrt{2}\theta {x}_{2}+{\theta }^{2}})]\\  &  & +\frac{1}{2}{k}_{2}{{x}_{2}}^{2}-(\frac{{b}_{2}{\theta }^{4}}{{\theta }^{4}+{{x}_{0}}^{4}}+{a}_{2}){x}_{2}\end{array}$$

It is found that the potential function equation (9) is a bistable system with the given parameter values. Then, the probability distribution *P*(*x*_0_, *x*_2_, *t*) of expression concentration of *X*_2_ obeys the following Fokker-Planck equation:

10 $$\frac{\partial P({x}_{0},{x}_{2},t)}{\partial t}=-\frac{\partial }{\partial {x}_{2}}[{F}_{2}({x}_{0},{x}_{2})P({x}_{0},{x}_{2},t)]+D\frac{{\partial }^{2}}{\partial {{x}_{2}}^{2}}P({x}_{0},{x}_{2},t)$$

By using the stationary solution of equation (10) and the steepest-descent approximation to equation (7), the MFPT can be given by

11 $$\tau =\frac{2\pi }{{|{U^{\prime\prime} }_{st}({x}_{0},{x}_{2}^{st}){U^{\prime\prime} }_{st}({x}_{0},{x}_{2}^{u})|}^{1/2}}\exp [\frac{{U}_{st}({x}_{0},{x}_{2}^{u})-{U}_{st}({x}_{0},{x}_{2}^{st})}{D}].$$

where ${x}_{2}^{st}$ and ${x}_{2}^{u}$ are the expression levels of gene *X*_2_ at the steady state and unstable steady state, ${U}_{st}({x}_{0},{x}_{2}^{st})$ and ${U}_{st}({x}_{0},{x}_{2}^{u})$ are the values of potential function at the steady state and unstable steady state, respectively.

## Results and Discussions

### Multiple phenotypes and phenotypic switching of a single breast cancer cell

In the last section, based on the regulatory mechanism of genes *zeb1* and *cdh1* in the growth and development of breast cancer cells, a general kinetic model was proposed. In this section, by using our kinetic model, it is shown that an individual breast cancer cell can exists in any of three possible phenotypic states (i.e., the stem-like, basal, and luminal states) which correspond to three basins of attractions of the probability landscape. The cell-state transition between the three states can be induced by the noise, and the properties of phenotypic switching are quantified by the mean first-passage time.

#### Deterministic trajectories, probability distribution, and potential landscape of model

**Figure 2:**
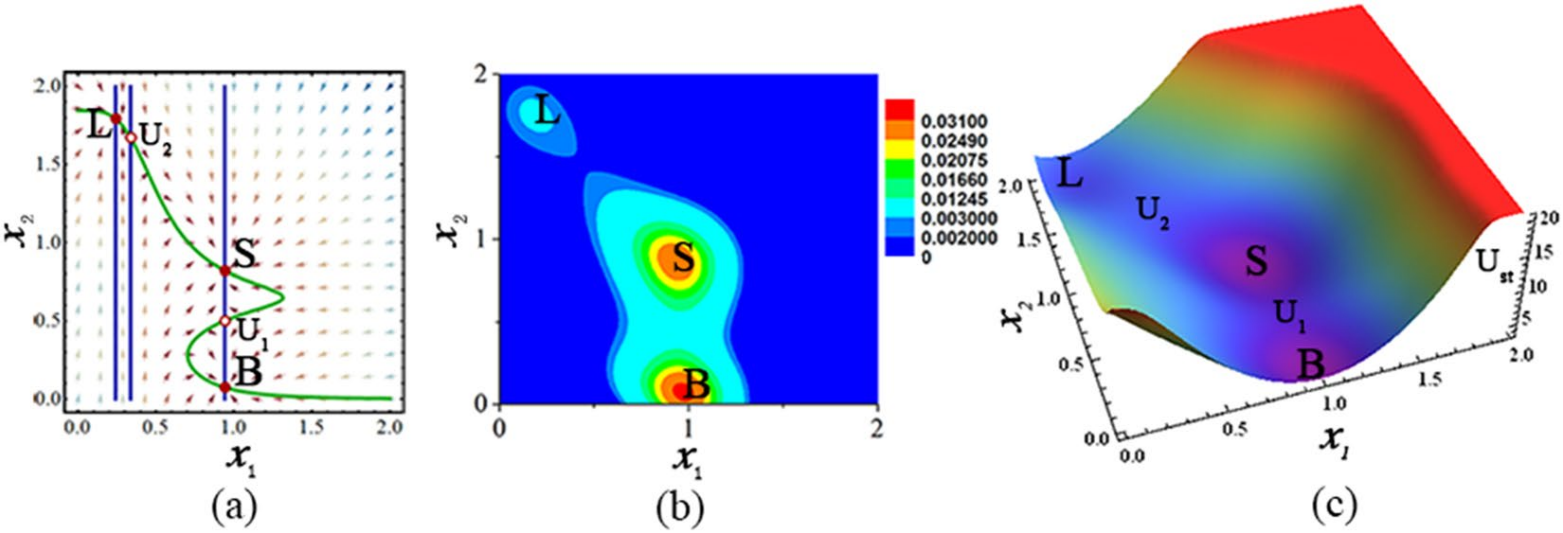
Deterministic trajectories, probability distribution, and potential landscape of model. (**a**) Deterministic trajectories of kinetic model for an individual cancer cell. There are three steady states (filled circles) corresponding to three phenotypic states (the stem-like (S), basal (B), and luminal (L) states), and two unstable steady states (circles, *U*_1_ and *U*_2_). (**b**) Effective landscape of stationary probability distribution *P*_*st*_(*x*_1_, *x*_2_) in two dimensions. (**c**) Potential landscape *U*_*st*_(*x*_1_, *x*_2_) in three dimensions. The other parameters: *a*_1_ = 0.8, *a*_2_ = 0.85, and *D* = 0.02.

By using the gene regulatory kinetic model, the multiple phenotypic states can arise in each breast cancer cell. Under the deterministic description equations (1) and (2), the deterministic trajectories of the kinetic model for each breast cancer cell show that there are three steady states and two unstable steady states as given by Fig. 2(a), and the three steady states correspond to the three phenotypes of each cancer cell: the stem-like (S), basal (B), and luminal (L) states.

Under the stochastic description of equations (3) and (4), the stationary probability *P*_*st*_(*x*_1_, *x*_2_) and the potential landscape *U*_*st*_(*x*_1_, *x*_2_) as given by Fig. 2(b,c) also show that a breast cancer cell can exist in any of three possible phenotypes (S, B, and L states) with different probabilities.

#### Multiple states coexistence and phase diagram

The variation of expression level of genes can be considered as the change of self-activation strength of genes. In our kinetic model, the self-activation strength *a*_1_ of transcription factor ZEB1 can be induced by the microenvironmental signal (e.g., the TGF-β signal)^51^, the expression level of protein E-Cadherin determined by self-activation strength *a*_2_ of gene *cdh1* can indicate the different stages of cancer. The expression of E-Cadherin in the early stage of some tumors is low (through allelic loss and methylation/hyper-methylation of 5’CpG sites of *cdh1*), while the expression is high in the late stage of the tumor^46–48^.

**Figure 3:**
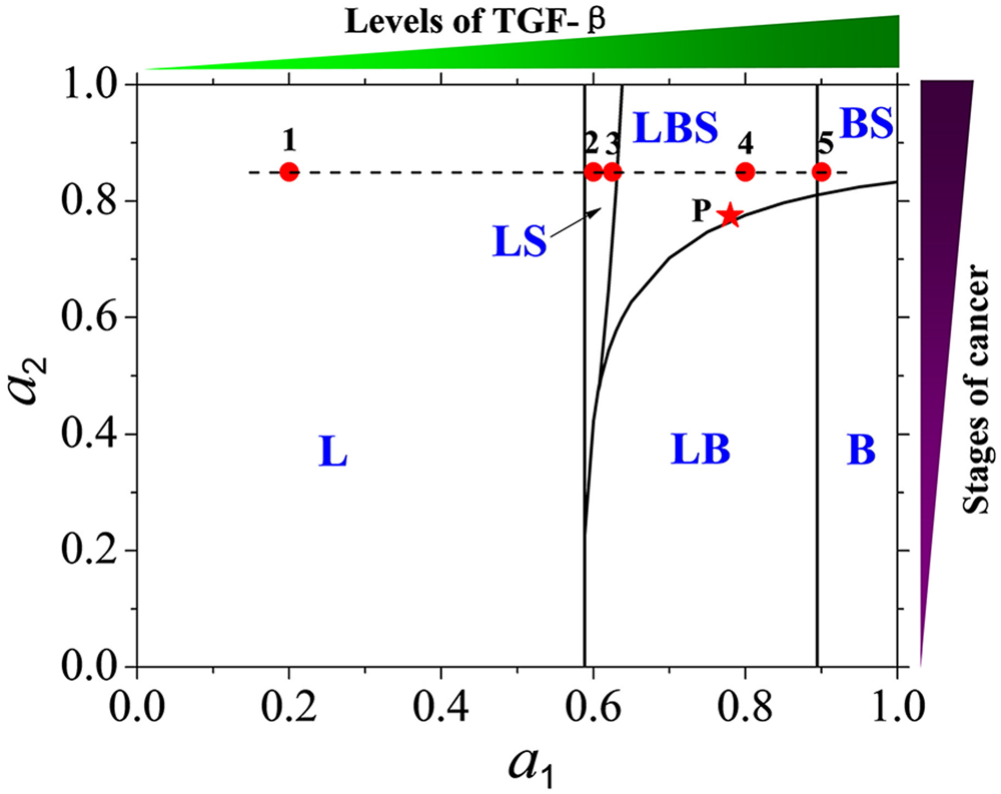
Phase diagram of multiple phenotypic states of a breast cancer cell in parameters (*a*_1_, *a*_2_) plane. The self-activation strength *a*_1_ of gene *zeb1* can be induced by the TGF-β signal, and the self-activation strength *a*_2_ of gene *cdh1* (encoding protein E-Cadherin) can indicate the different stages of cancers. The point P (the star) corresponds to phenotypic equilibrium distribution in the SUM159 breast cancer line^25^. The five points (1, 2, 3, 4, 5) on the dashed line correspond to the five clinical subtypes of breast cancer cells^43^.

A steady state of the kinetic model corresponds to a phenotypic state of a cancer cell. The steady state properties of the kinetic model show that there are the mono-stability (e.g., L or B), the bi-stability (e.g., LS, LB, or BS), and the tri-stability (e.g., LBS) under the different conditions. A phase diagram for the properties of phenotypic states of a single cancer cell is drawn in parameters (*a*_1_, *a*_2_) plane as shown by Fig. 3.

With the variation of parameter *a*_1_(or *a*_2_), the phenotypic states of each cancer cell are very different. From the phase diagram, it can be found that, in the early stage of cancer (i.e., *a*_2_ is small), a cancer cell is found in the L state at low level of TGF-β signal, and in the B state at high level of TGF-β signal. In the late stage of cancer (i.e., *a*_2_ is large), however, a cancer cell is found in the multiple phenotypic states coexistence at high level of TGF-β signal, such as the LS, LB, LBS, and BS states.

#### Phenotypic switching between states due to noise

In the regions of multiple phenotypic states coexistence (e.g., the LS, LB, BS or LBS in Fig. 3), the cell-state transition can be induced by the noise, and the properties of phenotypic switching between phenotypic states are characterized by using the MFPTs (obtained by the theoretical formula equation (11) and the numerical simulation of stochastic process according to equations (3) and (4)). Furthermore, the barrier height of minima of potential function equation (9) can also be used to imply the properties of phenotypic switching, the height of barriers of two attractors (e.g., the B and S states) is defined by:

12 $${\rm{\Delta }}{U}_{{u}_{1}B}={U}_{{u}_{1}}-{U}_{B},{\rm{\Delta }}{U}_{{u}_{1}S}={U}_{{u}_{1}}-{U}_{S}.$$

where *u*_1_ is the saddle point between B and S states.

It is interesting to note that in equation (10) the dependence on the height of potential function between the steady state and unstable steady state is contained in the exponential factor. The higher the barrier height is, the larger the MFPT of the phenotype will be. The larger MFPT means that this phenotype is more difficult to switch to the other phenotype. Hence, the barrier heights of minima of potential function can also be used to imply the transition directionality of phenotypic switching. In the case of multiple phenotypic states coexistence, for example, the heights of barriers of two attractors (B and S states) are defined byequation (12).

**Figure 4:**
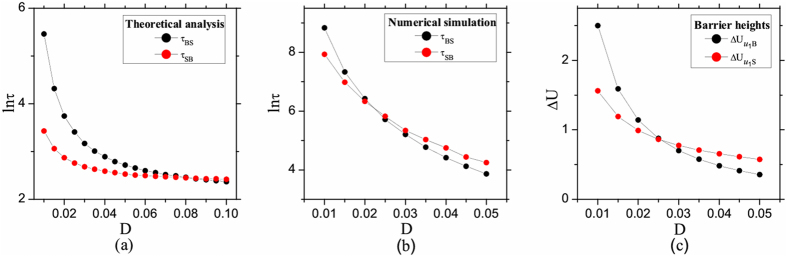
The properties of phenotypic switching due to noise in the multiple states coexistence regions. (**a**) The MFPTs (*τ*_*BS*_ and *τ*_*SB*_) are obtained by the theoretical formula equation (11). (**b**) The MFPTs (*τ*_*BS*_ and *τ*_*SB*_) are obtained by the numerical simulation of stochastic process of equations (3) and (4). (**c**) Relative barrier heights ${\rm{\Delta }}{U}_{{u}_{1}B}$ and ${\rm{\Delta }}{U}_{{u}_{1}S}$ are obtained by equation (12). The other parameters: *a*_1_ = 0.8, *a*_2_ = 0.85.

For instance, taking into account the interconversions between B and S states, Fig. 4 shows that both the MFPTs (obtained by the theoretical formula and the numerical simulation) and the barrier heights of minima (the B and S states) of potential function are decreased with the increasing of noise intensity *D*, and there exists a threshold (the cross point) of noise intensity when the phenotype of cancer cells converts between B and S states.

With the increasing of noise intensity *D*, Fig. 4(a) shows that *τ*_*BS*_ and *τ*_*SB*_ are decreased, and the threshold of noise intensity is *D*_*c*_ ≈ 0.085. When *D* < *D*_*c*_, *τ*_*SB*_ < *τ*_*BS*_, a cancer cell can change from S state to B state (i.e., *S* → *B*), and the cancer cell has much larger probability to stay in B phenotype. However, when *D* > *D*_*c*_, *τ*_*SB*_ > *τ*_*BS*_, a cancer cell can change from B state to S state (i.e., *B* → *S*), and the cancer cell has larger probability to stay in S phenotype.

It should be pointed out that, in the regions of multiple phenotypic states coexistence, the transitions between cell phenotypes can also be induced by the self-activation strength *a*_1_ of ZEB1 through the increasing of the TGF-β signal, the self-activation strength *a*_2_ of *cdh1* through the demethylation of 5′ CpG sites of *cdh1*, and the repression strength *b*_2_ of *cdh1* by ZEB1, respectively. Those data are not provided in this paper.

### Phenotypic equilibrium in SUM159 cell line

The SUM159 populations are composed of a large number of breast cancer cells, in which the kinetics of genes regulatory mechanisms in each cancer cell is identical. It was found that^25^ the phenomenon of phenotypic proportions in breast cancer cell lines is due to the interconversions between states. The multiple phenotypic states of each cancer cell at the level of single cancer cell can be mapped onto the macroscopic phenotypic equilibrium in subpopulations of cancer cells at the level of the whole cancer population, where the expression levels of genes *zeb1* and *cdh1* associated with different subpopulations are different.

#### Expressions level of genes *zeb1* and *cdh1* in SUM159 cell line

In SUM159 sorted cell subpopulations, the quantitative RT-PCR showed that (see the Fig. 1E in ref. 25) the expression level of gene *cdh1* (E-Cadherin) associated with stem and luminal states are specifically high, and the expression level of gene *cdh1* associated with basal state is low. However, the expression level of gene *zeb1* associated with stem state is same as that associated with basal state, and the expression level of gene *zeb1* associated with luminal state is lower than that associated with basal or stem state.

By utilizing our kinetic model, under the deterministic description, Fig. 2(a) shows that the relative expression levels of genes *zeb1* (i.e., *x*_1_) and *cdh1* (i.e., *x*_2_) at the three phenotypic states of each breast cancer cell are consistent with the experimental data of quantitative RT-PCR of genes associated with the stem-like, basal, and luminal states in SUM159 line (see the expression levels of Zeb1 and E-Cadherin in Fig. 1E of ref. 25).

#### Phenotypic equilibrium in SUM159 cell line

Under certain conditions (for example, at point P in the phase diagram Fig. 3), the cell-state equilibria in subpopulations of cancer cells^25^ can be explained by our stochastic kinetic model.

**Figure 5:**
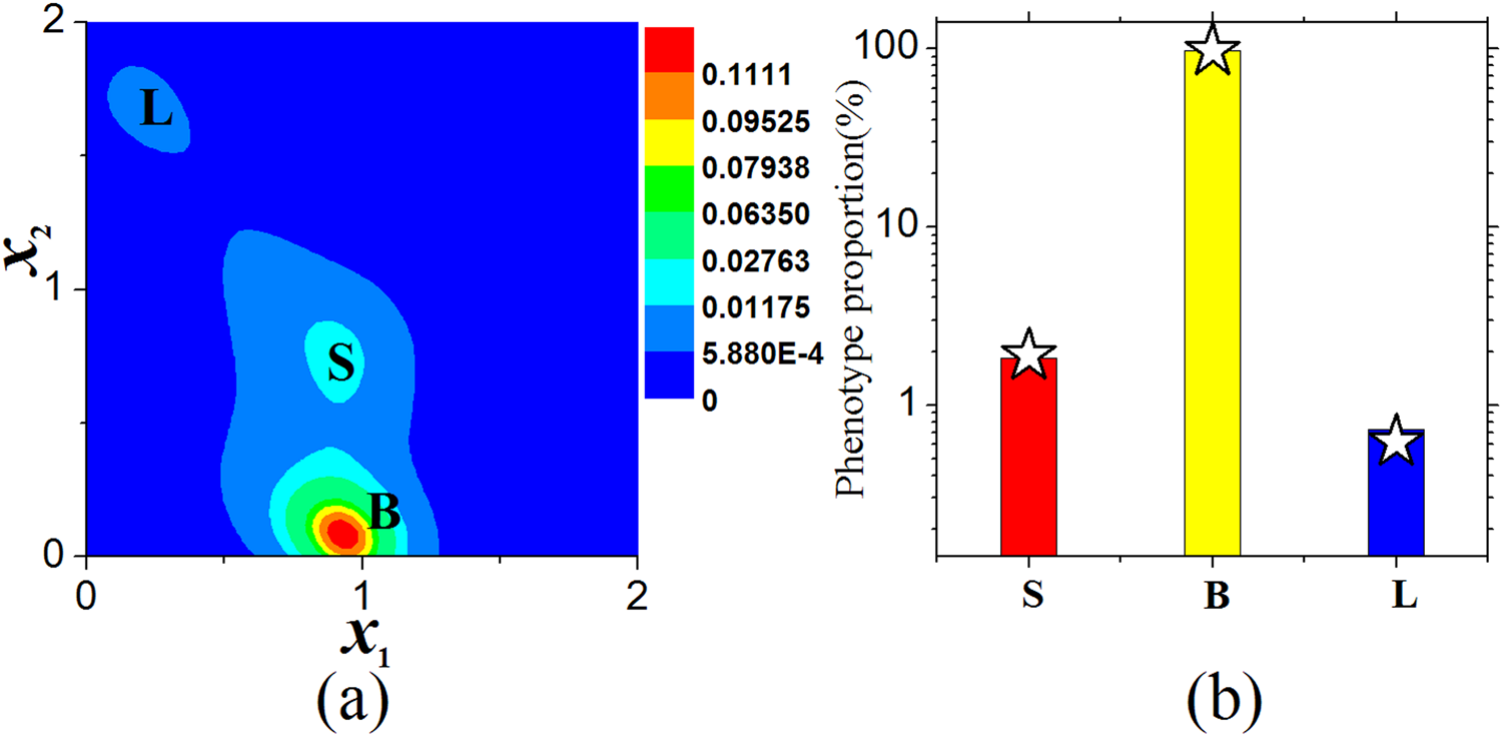
Phenotypic equilibrium in SUM159 line. (**a**) Effective landscape of stationary probability distribution *P*_*st*_(*x*_1_, *x*_2_) of three states (corresponding to point P in Fig. 3 with noise intensity *D* = 0.01) of an individual breast cancer cell. (**b**) Comparison the stationary probabilities proportions of three states (columns) of an individual cancer cell with the phenotypic equilibrium (stars represent the experimental data) in subpopulations of cancer cells in SUM159 line^25^.

Figure 5(a) shows the probability distributions of three phenotypes of a single cancer cell under certain noise intensity. Figure 5(b) shows that the cell-state proportions of three states of each cancer cell are consistent with those of phenotypic equilibrium in subpopulations of cancer cells (see the experimental data in Fig. 2B of ref. 25). The probability distribution is independent of the initial phenotype of each cancer cell, but depends on the microenvironmental fluctuations.

**Figure 6:**
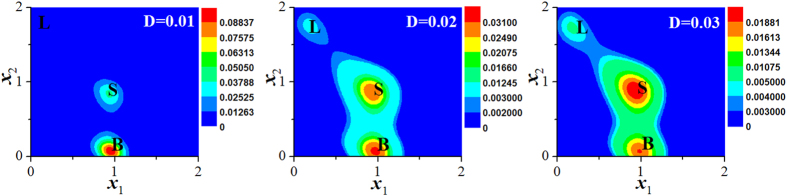
Effects of noises on phenotypic switching in the region of LBS coexistence. When noise intensity *D* = 0.01, the stationary probability distribution of L state is too small to visualize. The other parameters: *a*_1_ = 0.8 and *a*_2_ = 0.85.

Our stochastic model can also predict that, with the increasing of noise intensity, the probabilities of each cancer cell in both L and S states become large, yet that of each cancer cell in B state becomes small as shown in Fig. 6.

### Some macroscopic physiological phenomena of breast tumors

“Most cancer patients die from their disease as a result of metastasis”^57^. Cancer cells in distinct phenotypic states exhibit differences in functional properties. In this section, it is showed that some macroscopic phenomena of breast cancer cells at the level of the whole cancer populations can also be qualitatively understood by using of the microscopic genes regulatory kinetic model at the level of single cancer cell.

#### The “TGF-β paradox” in breast tumor therapy

TGF-β is a multifunctional cytokine, and plays an essential role in modulation of cellular growth, maturation, differentiation, apoptosis, adhesion, and microenvironment. In tumor therapy, the effects of TGF-β on cancer cells are quite different.

In the early stage of cancers, it can induce the epithelial cell cycle arrest and promote apoptosis through its canonical signaling pathway via SMAD protein. In the late stage of cancers, however, it is linked with supporting cancer progression, such as higher cell motility, cancer metastasis, and immune evasion through the non-canonical signaling pathway. These contrasting, dichotomous TGF-β behaviors in cancer development and progression are referred to as the “TGF-β paradox”^31–41^.

In fact, the contrasting, dichotomous TGF-β behaviors in the breast tumor therapy can be easily understood by using the phase diagram (Fig. 3) and the differences in functional properties of cancer cells within distinct phenotypic states.

In the early stage of cancers (i.e., *a*_2_ is small), with the increasing of TGF-β, Fig. 3 shows that a cancer cell can transform from one phenotypic state (L) to the multiple phenotypic states coexistence (LB, or LB and LS), and then to another one phenotypic state (B). When the cancer cell is in L phenotype, it stays in a tumor, in which it cannot be identified and killed by immune cells located around that tumor. With the increasing of the TGF-β level, the cancer cell is possible to switch to B state which has larger motility and can be identified and killed by the immune cells. Thus, the increasing of TGF-β can inhibit tumor metastasis in the early stage of cancers.

However, in the late stage of cancers (i.e., *a*_2_ is large), with the increasing of TGF-β signal, Fig. 3 shows that a cancer cell can transform from one state (L) to a successive multiple states coexistence (LS, LBS, and BS) process. In these coexisting states, the cancer cell has a certain probability of transition into S state, where it has the tumor-seeding ability, drug resistance, and the greatest cancer initiating capacity. Thus, the increasing of TGF-β can promote cancer progression in the late stage of cancers.

#### The five clinical subtypes of breast cancer cells

It was observed that there are three types of cancer stem-like cells (CSCs) in breast cancers (the basal, luminal, and basal-luminal CSCs)^42^. The basal CSCs are the mesenchymal-like state which comes from EMT, the luminal CSCs are the epithelial-like state which comes from MET, and the basal-luminal CSCs in which the two surface makers of both basal and luminal CSCs are simultaneously expressed.

More recently, Brooks *et al*.^43^ found that these three phenotypes steady proportion distributions were apparently different between the five clinical breast cancer subtypes which are luminal A, luminal B, Her2 positive, basal-like, and triple negative (TN, the clinically aggressive claudin-low subtype). The breast cancer clinical classification is based on the cellular surface markers expression of estrogen and progesterone receptor as well as the growth factor receptor HER2. The TN is similar to basal-like, which are both the most difficult ones to cure^58, 59^.

Our kinetic model of an individual cancer cell can be used to illustrate the five clinical subtypes of breast cancer cells since three types of CSCs have a similar pattern of gene expression and share a common regulatory pathway^42, 43^.

**Figure 7:**
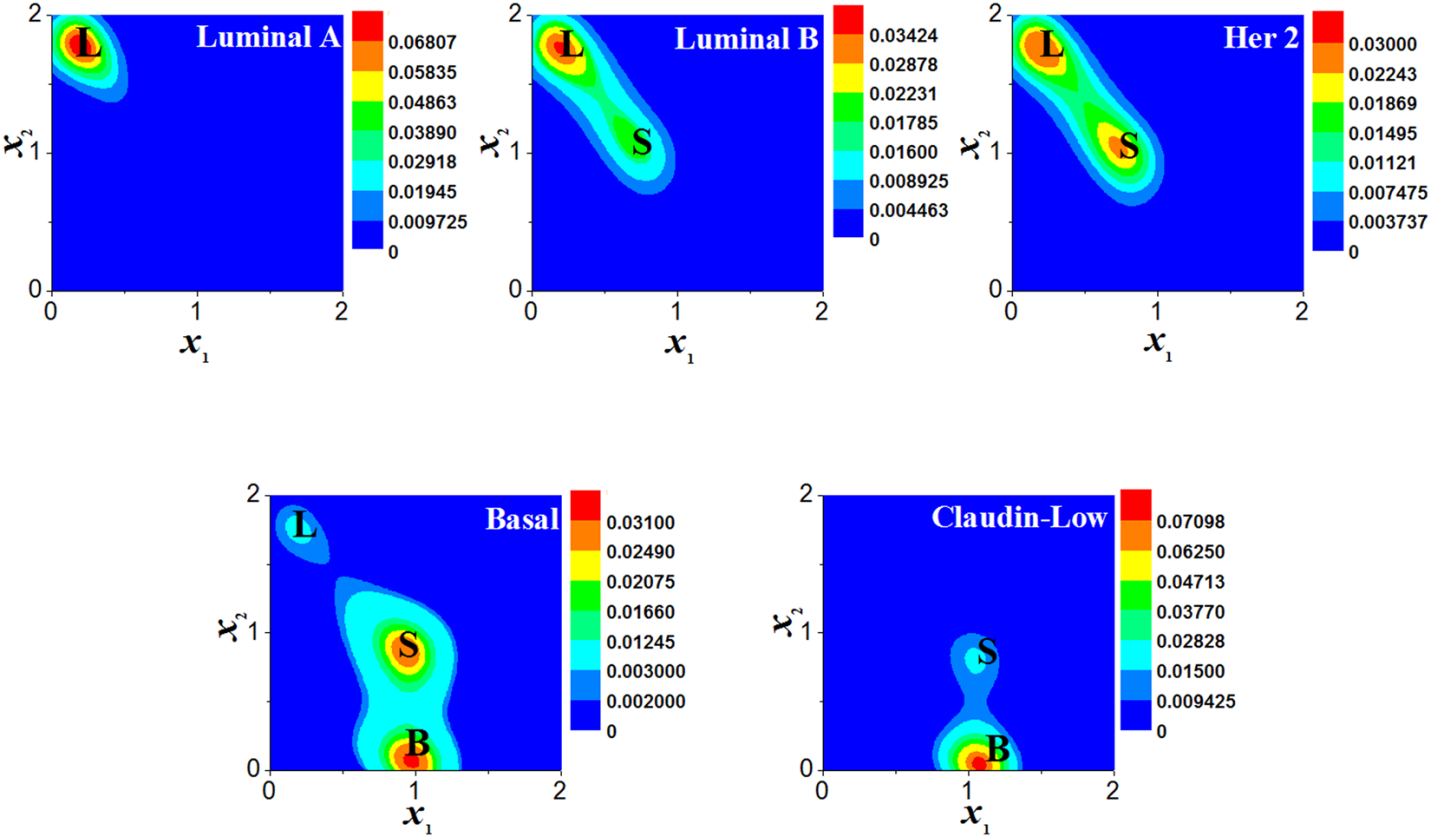
Effective landscape of stationary probability distributions of the three phenotypes. The probability distributions *P*_*st*_(*x*_1_, *x*_2_) corresponding to the five points 1, 2, 3, 4, and 5 in Fig. 3 with noise intensity *D* = 0.02 are similar to those of the five clinical subtypes (Luminal A, Luminal B, Her2, Basal, and Claudin-Low) of breast cancer^43^.

For each breast cancer cell, it is found that the five subtypes observed by clinical trials could be respectively corresponded to the five points 1, 2, 3, 4, 5 in the phase diagram of phenotypes (Fig. 3) in the late stage of cancers. The probability distribution of the five points are shown in Fig. 7, which are similar to those of clinical classification of breast cancers (see the Fig. 2 of ref. 43).

#### The effects of transient TGF-β on cancer metastasis

It was demonstrated that^44^ the consecutive high level of TGF-β can enhance the motility and intravasation of breast cancer cells by switching from cohesive to single cell motility but with low efficiency in forming new tumors at distant organs like the lung. However, the transient expression TGF-β can induce subsequent new tumor growth in the lungs. Thus, localized and reversible TGF-β signaling switches breast cancer cells from single cell motility to cohesive.

**Figure 8:**
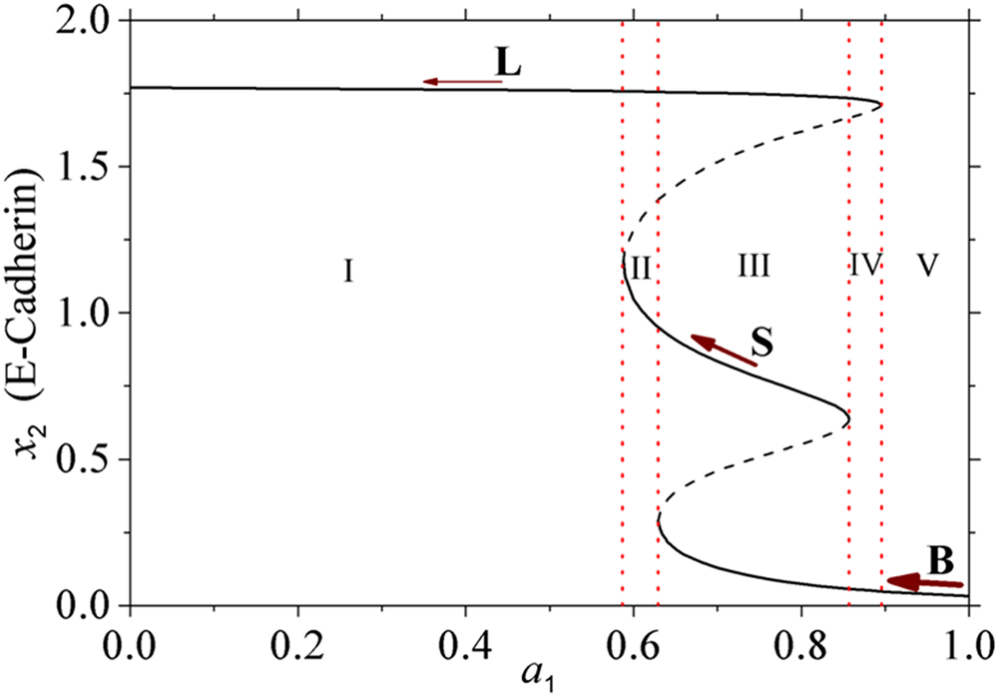
Bifurcation diagram of the expression level *x*_2_ of E-Cadherin as a function of *a*_1_. Parameter *a*_1_ represents the TGF-β signal since the self-activation strength *a*_1_ of gene *zeb1* can be induced by the TGF-β signal. The motility capacity of cancer cells is decreased step by step as shown by the size of arrow in different regions. The parameter: *a*_2_ = 0.8.

By virtue of the kinetic model, in the late stage of cancers (e.g., *a*_2_ = 0.8), when TGF-β signal is consecutively at high level (e.g., *a*_1_ > 0.9), Fig. 8 shows that the cancer cell is in the B state, in this case, the motility of the cancer cell is enhanced by the consecutive increasing of TGF-β.

**Figure 9:**
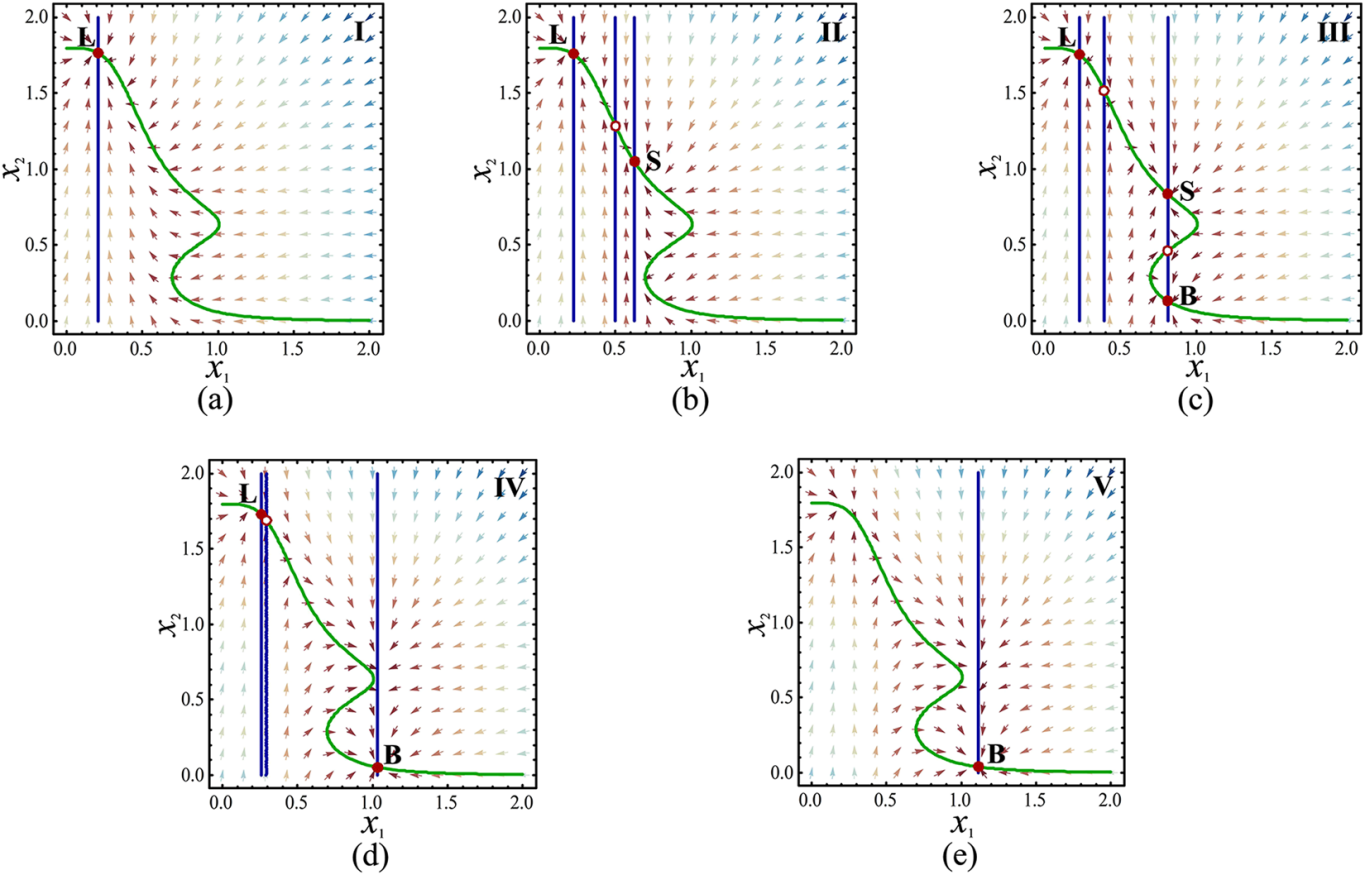
Deterministic trajectories of the five regions (I-V) in Fig. 8. The filled circle corresponds to the stable state, and the circle corresponds to the unstable steady states. (**a**) *a*_1_ = 0.3. (**b**) *a*_1_ = 0.6. (**c**) *a*_1_ = 0.7. (**d**) *a*_1_ = 0.88. (**e**) *a*_1_ = 0.95.

However, when TGF-β is instantaneously increased to a high level (e.g., *a*_1_ > 0.9) and decreased subsequently, with the decreasing of TGF-β, Fig. 8 shows that a cancer cell converts from B (Fig. 9(e)) state to LB (Fig. 9(d)), LBS (Fig. 9(c)), LS (Fig. 9(b)) states, and ends in L (Fig. 9(a)) state, respectively. In this process, the cancer cell gradually loses the ability to metastasize since the cancer cell can transfer into S or L state with a certain probability, and more and more of cancer cells have the ability to stick to distant organs and become resistant to immune cells, radiotherapy, and chemotherapy.

The motility capacities of cancer cells is decreased step by step as shown by the size of arrows in Fig. 8. The consecutive high expression of TGF-β can enhance the metastasis ability through promoting EMT, but the transient expression of TGF-β can induce the reversible process MET, which makes it easier for the cancer cells to stick to new sites through enhancing the expression of E-Cadherin, and form new tumors.

## Conclusions and Discussions

In this paper, a general kinetic model of microscopic regulatory mechanisms between two genes (*zeb1* and *cdh1*) with a transcriptional negative regulation at the level of single cancer cell was proposed to uncover several interesting macroscopic physiological phenomena of cancer cells observed by experiments and clinical trials, such as the phenotypic equilibrium in populations of breast cancer cell lines, the “TGF-β paradox” in tumor therapy, the five clinical subtypes of breast cancer cells, and the effects of transient TGF-β on cancer metastasis.

By using the effective landscape through construction of underlying stationary probability, it is found that each breast cancer cell can also exist in any of three possible phenotypic states (i.e., the stem-like, basal, and luminal states) which correspond to three basins of attractions of the probability landscape. The transitions between the three states are induced by the noise (or the self-activation strength, or the repression strength of genes). The property of phenotypic switching is quantified by the mean first-passage time. Under certain conditions, the probabilities of each breast cancer cell appearing in the three states are consistent with the macroscopic phenotypic equilibrium in subpopulations of breast cancer cells observed by the experiments^25^. Comparing with the Markov model^25^ proposed by Gupta *et al*. which is a macroscopic model, our kinetic model which depends on microscopic regulatory mechanisms between two genes is more fundamental and can explain more physiological phenomena of breast cancers observed by experiments and clinical trials.

The phase diagram of the deterministic kinetic model in parameters (*a*_1_, *a*_2_) plane given by Fig. 3 shows that there are multiple phenotypic states coexistence regions (e.g., LB, LBS, LS, BS). The five breast cancer clinical subtypes^42, 43^ can be explained by different proportion distributions of three cell phenotypes of each cancer cell, and the “TGF-β paradox”^31–41^ in the tumor therapy can be understood by the phase diagram. With the increasing of TGF-β signal, the motility of cancer cells is increased. While the motility of cancer cells is decreased by decreasing TGF-β, then the cancer cells can reach and form new tumors at distant organs^44^. Thus, high level of TGF-β signal is worse prognosis for tumors in the late stage of cancers.

In order to broadly explore the parameters used in the model, we first drew different phase diagrams with different parameters combinations, such as (*a*_1_, *b*_2_),(*b*_1_, *a*_2_) etc., and found they all had the similar phase diagrams which contain the same multiple phenotypic states coexistence regions (e.g., LB, LBS, LS, BS). Second, we changed the Hill power parameter *n* and found that there always exist three steady states which correspond to the stem-like, basal, luminal states except *n* < 2.24.

Our kinetic model also predicts that there exists a threshold of noise intensity when the phenotype of a cancer cell transits between B state and S state. Due to the complexities of the equations which is highly nonlinear and have two unknowns, it is difficult to calculate the corresponding potential functions between any two states, such as L and B or L and S, except B and S states which coincidentally have the same value of x_1_. However, the role of noise on state conversions is also numerically studied between the states of L, B and L, S in the LBS region and we found that higher noise intensity induce the cancer cell state switching from L to B or S state which means enhancing noise intensity can promote the breast cancer metastasis. Although our model can reveal the multiple phenotypic states and phenotypic switching of breast cancer cells, it also should be mentioned that the real regulatory network of cell phenotype decisions is much more complex and there are probably other genes taking part in the dynamics of phenotypic switching. Above results reveal that the increasing of TGF-β can promote the metastasis ability of tumors through the EMT process, whereas the enhancing of the E-Cadherin expression, the noise intensity, and the transitory TGF-β signal can induce the forming of new tumors at distant organs through the opposite process MET.

In conclusion, by using a general kinetic model of microscopic regulatory mechanisms between two key genes, we demonstrated that the multiple phenotypic states of each cancer cell at the level of single cancer cell can be mapped onto some macroscopic physiological phenomena of breast cancer cells observed by experiments and clinical trials. Our results could provide new insights into the roles of microenvironmental signals and fluctuations at different stages of cancer cells, and the kinetic model might give some insights for various tumors clinical therapy strategies.
